# Association of changes and cumulative measures of triglyceride-glucose index-body mass index with hypertension risk: a prospective cohort study

**DOI:** 10.1186/s12889-024-20154-z

**Published:** 2024-09-27

**Authors:** Jiamin Yan, Min-zhe Zhang, Qi-qiang He

**Affiliations:** 1 Department of Laboratory Medicine, Wuhan Children’s Hospital (Wuhan Maternal and Child Healthcare Hospital), Huazhong University of Science & Technology, Wuhan, P. R. China; 2 School of Public Health, Wuhan University, Wuhan, China; 3 Hubei Biomass-Resource Chemistry and Environmental Biotechnology Key Laboratory, Wuhan University, Wuhan, China

**Keywords:** Hypertension, TyG-BMI, CHARLS, Cohort study

## Abstract

**Background:**

To investigate the relationships of the dynamic changes in triglyceride glucose index-body mass index (TyG‑BMI) and cumulative TyG-BMI with the risk of hypertension among middle-aged and elderly Chinese.

**Methods:**

Data were used from the China Health and Retirement Longitudinal Study (CHARLS). Participants who participated in the baseline study (2011–2012) and in subsequent surveys (2015–2018) were included in this study. The primary exposures were changes in TyG-BMI and cumulative TyG-BMI from 2012 to 2015. Changes in TyG-BMI were categorized using k-means clustering methods, while cumulative TyG-BMI was categorized into quartiles. Cox proportional hazards regression models were performed to examine the association between changes in TyG-BMI and cumulative TyG-BMI with the incidence of hypertension. Linear regression analyzes were performed to examine the association between changes in TyG-BMI and cumulative TyG-BMI with cumulative systolic blood pressure (SBP) and cumulative diastolic blood pressure (DBP).

**Results:**

Of a total of 2,561 participants aged 56.93 ± 8.08 years old at baseline, 253 individuals (9.9%) developed hypertension during the 7-year follow-up period. The hazard ratios (HR) and 95% confidence interval (CI) for hypertension were 1.50 (1.10–2.03) for class 2 (persistently medium class) and 2.35 (1.61–3.42) for class 3 (persistently high class), compared to class 1 (persistently low class). Additionally, class 2 showed increases of 7.70 mmHg (95% CI: 5.18–10.21) in cumulative SBP and 6.53 mmHg (95% CI: 4.68–8.38) in cumulative DBP, while class 3 exhibited increases of 14.10 mmHg (95% CI: 10.56–17.64) in cumulative SBP and 12.64 mmHg (95% CI: 10.03–15.25) in cumulative DBP, compared with class 1. Regarding cumulative TyG-BMI, the HR for hypertension were 1.75 (95% CI: 1.18–2.59) for quartile 3 and 2.15 (95% CI: 1.43–3.23) for quartile 4, compared with quartile 1. In quartile 2, cumulative SBP increased by 3.99 mmHg (95% CI: 0.88–7.11) and cumulative DBP by 2.74 mmHg (95% CI: 0.45–5.02). Quartile 3 showed increases of 8.32 mmHg (95% CI: 5.09–11.54) in cumulative SBP and 7.13 mmHg (95% CI: 4.76–9.49) in cumulative DBP. Quartile 4 exhibited the highest increases, with cumulative SBP rising by 13.15 mmHg (95% CI: 9.70–16.60) and cumulative DBP by 12.20 mmHg (95% CI: 9.67–14.74). Furthermore, a linear relationship was observed between cumulative TyG-BMI and the risk of hypertension.

**Conclusions:**

Changes in TyG-BMI and cumulative TyG-BMI were associated with an increased risk of hypertension, as well as higher cumulative SBP and DBP in Chinese middle-aged and elderly population.

## Introduction

Hypertension, a leading risk factor for cardiovascular disease, is a major public health concern globally. In 2019, the global prevalence of hypertension among adults aged 30–79 years was 1.28 billion, with two-thirds of cases concentrated in low- and middle-income countries, and hypertension is responsible for about one-fifth of all deaths worldwide [1–3]. In China, the prevalence of hypertension has increased significantly over the past 30 years. According to the Chinese National Hypertension Survey, it increased from 11.3% in 1991 to 23.2% in 2012–2015, resulting in approximately 270 million hypertensive patients [4, 5]. Furthermore, the Global Burden of Disease (GBD) study has shown that hypertension accounts for the largest proportion of disability-adjusted life years in China and is responsible for 24.6% of all-cause mortality [6]. Given this substantial public health burden, it is crucial to early identify risk factors to prevent hypertension and implement timely interventions.

Insulin resistance (IR), marked by impaired glucose metabolism in skeletal muscle, adipose tissue, and the liver, is a key factor in atherosclerosis and hypertension [7, 8]. While the hyperinsulinemic-euglycemic clamp (HEC) is the gold standard for detecting IR [9], its complexity and cost make it impractical for routine use. This highlights the need for a reliable and accessible alternative to assess IR widely. Therefore, simpler indices like triglyceride-glucose index (TyG), which has demonstrated high efficacy in detecting IR, are essential for widespread clinical application [10].

Recent studies have suggested a potential link between TyG combined with body mass index (BMI), termed TyG-BMI, and the risk of hypertension [11, 12]. While the TyG index is sensitive to insulin resistance, it may underestimate metabolic risk in individuals with significant obesity but normal triglyceride and glucose levels. Conversely, BMI alone does not account for differences in insulin sensitivity among individuals with similar BMI. By combining these two indices, the TyG-BMI index improves the ability to identify individuals at higher risk of metabolic disorders, providing a more comprehensive assessment of metabolic health than TyG [13, 14]. However, previous studies on TyG-BMI and hypertension have mostly focused on single TyG-BMI measurement. Investigation on the association between the dynamic change of TyG-BMI and hypertension risk is limited. A recent study by Liu et al. examined the association between changes in the TyG-BMI index (calculated as the 2015 TyG-BMI minus the baseline TyG-BMI) and the risk of developing hypertension [15]. However, this study used hypertension status in 2015 as the outcome, which may compromise the accuracy in assessing the causal relationship. Additionally, the study only investigated four years and did not examine cumulative TyG-BMI and its effects on blood pressure. Herein, we reclassified the changes in TyG-BMI using a machine learning approach (k-means clustering) to investigate the effects of changes in TyG-BMI and cumulative TyG-BMI on the incidence of hypertension in middle-aged and elderly Chinese with a 7-year follow-up, providing new strategies for the prevention and treatment of hypertension in this population.

## Methods

### Study population

We utilized data from the China Health and Retirement Longitudinal Study (CHARLS), a nationally representative longitudinal study examining the health and economic conditions of individuals aged 45 and older in China. CHARLS was conducted on individuals from 450 rural and urban communities across China every two years. The baseline survey was conducted in 2011–2012, and to date, there have been five rounds of CHARLS (2011, 2013, 2015, 2018, and 2020). CHARLS received approval from the Biomedical Ethics Committee of Peking University (IRB00001052–11015), and all participants provided signed consent.

**Fig. 1:**
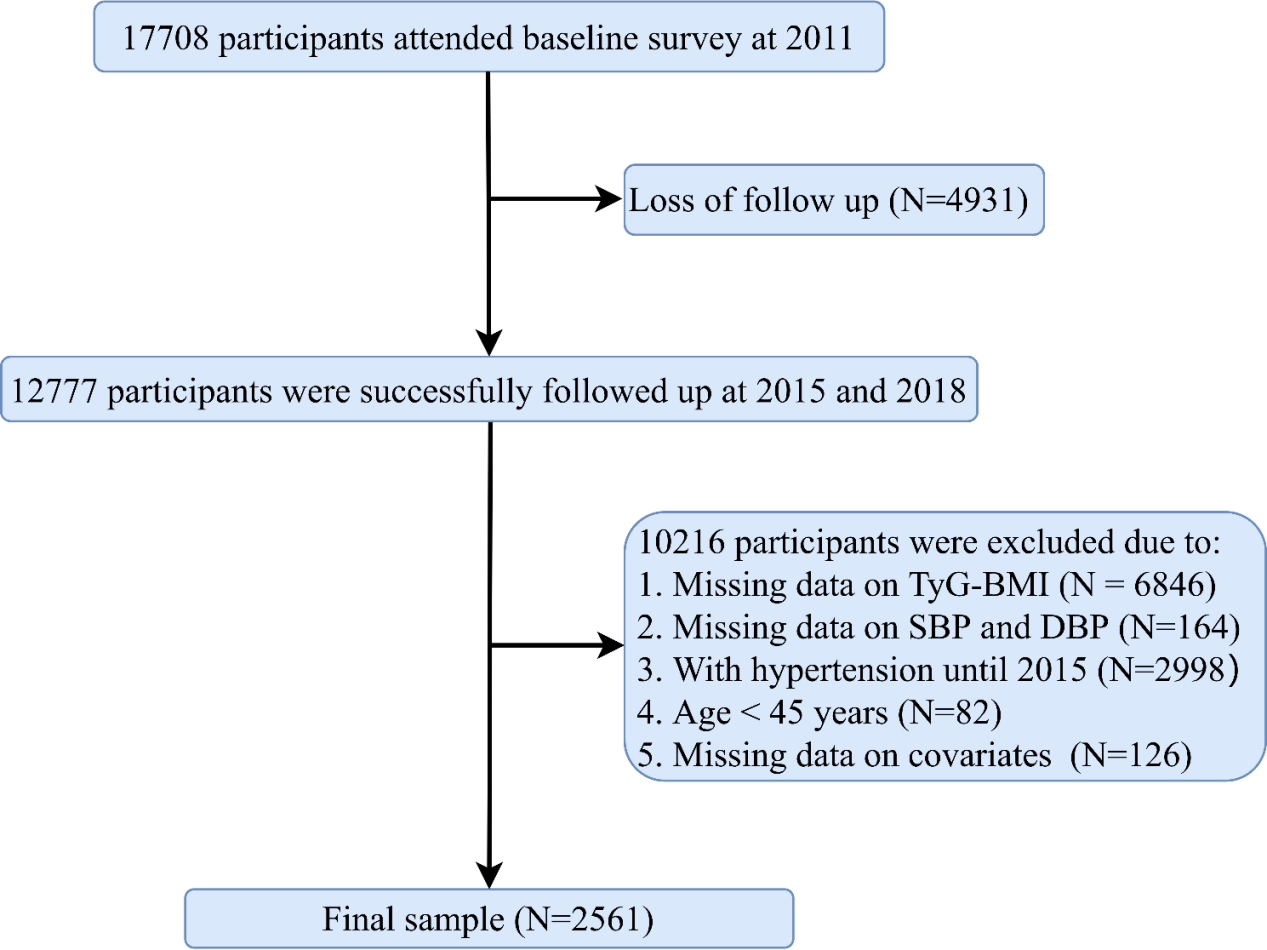
Flow chart of the study population

As the measurement of blood samples was only conducted in CHARLS 2011–2012 and 2015, therefore the present study included participants who took part in 2011–2012 (baseline), 2015, and 2018. Of the 17,708 participants, we excluded 4,931 participants who were lost to follow-up, 7,010 participants without data on TyG, BMI, diastolic blood pressure (SBP), and systolic blood pressure (DBP), 2,998 participants with hypertension before 2018, 82 participants aged < 45 years, and 126 participants with missing data on covariates such as sex, education level, etc. Finally, a total of 2,561 participants were included in the data analysis (Fig. 1).

### Exposures and outcomes

The TyG-BMI was calculated using the following formula: Ln [triglyceride (TG) (mg/dL) × fasting plasma glucose (FBG) (mg/dL)/2] × BMI. Serum TG and FBG were examined at the Youanmen Center for Clinical Laboratory of Capital Medical University using enzymatic colorimetric test. The cumulative TyG-BMI was calculated using the following formula: (TyG-BMI_2012_ + TyG-BMI_2015_)/2 × (2015 − 2012) [16]. The cumulative SBP and DBP was calculated using the following formula: (SBP_2012_ + SBP_2015_)/2 × (2015 − 2012), (DBP_2012_ + DBP_2015_)/2 × (2015 − 2012), respectively [17]. Hypertension was defined as new-onset hypertension that occurred during the follow-up period. Hypertension was defined as SBP ≥ 140 mmHg, or DBP ≥ 90 mmHg, or diagnosed with hypertension by a doctor, or current use of antihypertensive medication [18].

### Covariates

In lines with the Chinese Hypertension Guidelines and previous studies on hypertension in China, we included covariates including sex, age (< 60 years and ≥ 60 years), education level (illiterate, primary school and middle school or above), marital status (married and single or other), residence (rural and urban), smoking status (smoking and non-smoking), alcohol drinking (> 1 times per month, < 1 times per month and non-consumer), history of dyslipidemia, diabetes, heart disease, stroke, and kidney disease, and CRP. The laboratory examination included tests for glycosylated hemoglobin type A1c (HbA1c), total cholesterol (TC), high-density lipoprotein cholesterol (HDL-C), low-density lipoprotein cholesterol (LDL-C), and C-reactive protein (CRP). Estimated glomerular filtration rate (eGFR) was calculated using CKD-EPI Chronic Kidney Disease Epidemiology Collaboration (CKD-EPI) 2021 formula [19]. Heart disease was defined by either diagnosis from a doctor or the administration of treatment. Stroke was defined as being diagnosed by a doctor or receiving treatment for stroke. Dyslipidemia was characterized by TC level of ≥ 240 mg/dL, TG level of ≥ 150 mg/dL, LDL-C level of ≥ 160 mg/dL, HDL-C level of < 40 mg/dL, or ongoing lipid-lowering treatments, or a medical diagnosis [20]. Diabetes was defined as FBG level ≥ 126 mg/dl, or HbA1c level ≥ 6.5%, or antidiabetic medication usage, or a medical diagnosis [21]. Kidney disease was defined as eGFR level < 60 ml/min/1.73m^2^, or receiving treatment for kidney issues, or diagnosis by a doctor [22].

### Statistical analysis

The changes in TyG-BMI were classified using the k-means clustering method, implemented with the stats and factoextra packages. K-means clustering is a widely used unsupervised learning algorithm to partition a dataset into distinct clusters, where each data point is assigned to the cluster with the nearest mean (centroid) [23, 24]. The algorithm minimizes the sum of squared Euclidean distances between data points and their corresponding cluster centroids, thus optimizing clustering. To determine the optimal number of clusters, both the elbow method and the gap statistic were employed. The elbow method was performed by clustering for different values of k (typically between 1 and 10), calculating the total within-cluster sum of squares (WSS) for each k, and plotting the WSS against k. The “elbow” point, where the rate of WSS reduction drops sharply, indicates the optimal number of clusters. The gap statistic compares the total within-cluster variation for different k-values with that of reference datasets generated under a null hypothesis of random distribution. The optimal number of clusters is determined where the gap statistic reaches its maximum, signaling a significant difference between the observed clustering and random reference datasets. In our study, the value of the between-cluster sum of squares (BSS) divided by the total sum of squares (TSS) was 73.5%. This indicates that the clustering explained 73.5% of the total variance, suggesting a fairly successful clustering outcome.

Differences between the three classes of changes in TyG-BMI were assessed using one-way ANOVA or Kruskal-Wallis test for continuous variables, depending on whether the data met the prerequisite conditions (homogeneity of variance, normal distribution, and independence). For categorical variables, chi-square test or Fisher’s exact test was applied as appropriate. Cumulative TyG-BMI was divided into four categories based on the quartile. Three models were then created to examine the relationship between changes in TyG-BMI, cumulative TyG-BMI and hypertension risk using Cox proportional hazards regression models.

Model 1 was adjusted for age and sex. Model 2 was additionally adjusted for education level, marital status, residence, smoking, and alcohol drinking. Model 3 was additionally adjusted for dyslipidemia, diabetes, heart disease, stroke, kidney disease, and CRP based on model 2.

To evaluate the effectiveness of TyG-BMI in predicting hypertension, receiver operating characteristic (ROC) curve analysis was performed using the pROC package. The Delong’s test was performed to compare the predictive performance of TyG-BMI with that of TyG. In addition, restricted cubic spline (RCS) regression models with the rms package were used to examine the nonlinear relationship between cumulative TyG-BMI and hypertension. This model employed 4 knots positioned at the 5th, 35th, 65th, and 95th percentiles of cumulative TyG-BMI, with the reference set as the 5th percentile.

We next investigated the association of changes in TyG-BMI and cumulative TyG-BMI with cumulative SBP and DBP using linear models. These models were controlled for various confounders as described in above model 3. Finally, subgroup analyses were performed to assess the changes in TyG-BMI and cumulative TyG-BMI with hypertension risk according to participants’ characteristics. All analyses were performed using R (version 4.3.2). A two-sided *P* < 0.05 was considered statistically significant.

## Results

### Baseline characteristics of study participants

Table 1 shows the basic characteristics of the study participants according to the changes in TyG-BMI from 2012 to 2015. Among the 2,561 participants aged 56.93 ± 8.08 years old, there were 1,142 males (44.6%) and 1,419 females (55.4%). Their TyG-BMI was 195.83 ± 36.00 in 2012 and 199.00 ± 36.22 in 2015, with a mean cumulative TyG-BMI of 592.24 ± 102.41. The majority had a low level of education (67.1%), were married (92.6%), and lived in rural areas (69.3%). Additionally, 41.5% of the participants had dyslipidemia, 12.0% had diabetes, 7.6% had heart disease, 1.1% had experienced stroke, and 7.2% had kidney disease. Participants in class 2 and class 3 exhibited higher levels of HbA1c, TC, and LDL-C, as well as a higher prevalence of dyslipidemia and diabetes, compared to those in class 1.

**Table 1:** Baseline characteristics of participants according to changes in TyG-BMI

	Total (*N* = 2561)	Class 1 (*n* = 1050)	Class 2 (*n* = 1089)	Class 3 (*n* = 422)	*P* value
**Age (years**,** M ± SD)**	56.93 ± 8.08	58.64 ± 8.63	56.21 ± 7.55	54.50 ± 7.02	< 0.001
**Sex (%)**					< 0.001
Male	1142 (44.6)	575 (54.8)	427 (39.2)	140 (33.2)	
Female	1419 (55.4)	475 (45.2)	662 (60.8)	282 (66.8)	
**Education level (%)**					< 0.001
Illiterate	660 (25.8)	293 (27.9)	285 (26.2)	82 (19.4)	
Primary school	1058 (41.3)	461 (43.9)	422 (38.8)	175 (41.5)	
Middle school or above	843 (32.9)	296 (28.2)	382 (35.1)	165 (39.1)	
**Marital status (%)**					0.110
Married	2371 (92.6)	962 (91.6)	1009 (92.7)	400 (94.8)	
Single or other	190 (7.4)	88 (8.4)	80 (7.3)	22 (5.2)	
**Residence (%)**					< 0.001
Rural	1775 (69.3)	796 (75.8)	729 (66.9)	250 (59.2)	
Urban	786 (30.7)	254 (24.2)	360 (33.1)	172 (40.8)	
**Smoking (%)**					< 0.001
Smoking	959 (37.4)	492 (46.9)	343 (31.5)	124 (70.6)	
Non-smoking	1602 (62.6)	558 (53.1)	746 (68.5)	298 (29.4)	
**Alcohol drinking (%)**					< 0.001
> 1 times per month	631 (24.6)	308 (29.3)	240 (22.0)	83 (19.7)	
< 1 times per month	228 (8.9)	102 (9.7)	90 (8.3)	36 (8.5)	
Non-consumer	1702 (66.5)	640 (61.0)	759 (69.7)	303 (71.8)	
**Dyslipidemia (%)**					< 0.001
Yes	1064 (41.5)	241 (23.0)	508 (46.6)	315 (74.6)	
No	1497 (58.5)	809 (77.0)	581 (53.4)	107 (25.4)	
**Diabetes (%)**					< 0.001
Yes	307 (12.0)	81 (7.7)	131 (12.0)	95 (22.5)	
No	2254 (88.0)	969 (92.3)	958 (88.0)	327 (77.5)	
**Heart disease (%)**					0.228
Yes	194 (7.6)	72 (6.9)	82 (7.5)	40 (9.5)	
No	2367 (92.4)	978 (93.1)	1007 (92.5)	382 (90.5)	
**Stroke (%)**					0.680
Yes	29 (1.1)	10 (1.0)	13 (1.2)	6 (1.4)	
No	2532 (98.9)	1040 (99.0)	1076 (98.8)	416 (98.6)	
**Kidney disease (%)**					0.590
Yes	184 (7.2)	82 (7.8)	74 (6.8)	28 (6.6)	
No	2377 (92.8)	968 (92.2)	1015 (93.2)	394 (93.4)	
**HbA1c (mg/L**,** M ± SD)**	5.19 ± 0.66	5.12 ± 0.53	5.18 ± 0.65	5.42 ± 0.90	< 0.001
**Total cholesterol** **(mg/L**,** M ± SD)**	188.81 ± 35.70	182.04 ± 34.41	192.15 ± 35.15	197.05 ± 37.36	< 0.001
**HDL-C** **(mg/L**,** M ± SD)**	52.21 ± 14.89	57.84 ± 15.39	50.77 ± 12.91	41.92 ± 11.66	< 0.001
**LDL-C** **(mg/L**,** M ± SD)**	113.66 ± 32.35	108.79 ± 29.72	118.18 ± 31.95	114.09 ± 37.57	< 0.001
**eGFR (ml/min/1.73m**^**2**^, **M ± SD)**	95.01 ± 13.00	94.58 ± 12.73	95.02 ± 13.24	96.06 ± 12.98	0.142
**CRP (mg/L**,** M ± SD)**	2.23 ± 7.34	2.60 ± 10.31	1.88 ± 4.36	2.22 ± 3.60	0.074
**SBP**_**2012**_ **(** **mmHg**,** M ± SD)**	116.20 ± 11.17	114.81 ± 11.37	116.53 ± 10.98	118.82 ± 10.64	< 0.001
**SBP**_**2015**_ **(** **mmHg**,** M ± SD)**	116.91 ± 11.55	115.23 ± 12.12	117.57 ± 11.22	119.40 ± 10.28	< 0.001
**DBP**_**2012**_ **(** **mmHg**,** M ± SD)**	69.38 ± 8.61	67.62 ± 8.58	69.98 ± 8.43	72.21 ± 8.21	< 0.001
**DBP**_**2015**_ **(** **mmHg**,** M ± SD)**	69.86 ± 8.05	68.35 ± 8.17	70.36 ± 7.75	72.30 ± 7.75	< 0.001
**Cumulative SBP (** **mmHg**,** M ± SD)**	349.67 ± 28.89	345.05 ± 29.72	351.15 ± 28.01	357.33 ± 27.00	< 0.001
**Cumulative DBP (** **mmHg**,** M ± SD)**	208.85 ± 21.37	203.96 ± 21.23	210.51 ± 20.62	216.76 ± 20.64	< 0.001
**FBG**_**2012 **_**(mg/L**,** M ± SD)**	105.27 ± 27.72	100.95 ± 22.48	104.48 ± 22.21	118.08 ± 43.90	< 0.001
**FBG**_**2015**_** (mg/L**,** M ± SD)**	99.49 ± 30.07	93.41 ± 19.56	100.43 ± 29.63	112.21 ± 44.92	< 0.001
**TG**_**2012 **_**(mg/L**,** M ± SD)**	119.74 ± 87.84	87.34 ± 44.73	121.57 ± 72.86	195.61 ± 140.78	< 0.001
**TG**_**2015 **_**(mg/L**,** M ± SD)**	131.89 ± 83.58	93.25 ± 42.71	138.97 ± 76.33	209.73 ± 113.80	< 0.001
**BMI**_**2012 **_ **(M ± SD)**	22.81 ± 3.44	20.03 ± 1.74	23.53 ± 1.72	27.85 ± 3.27	< 0.001
**BMI**_**2015 **_ **(M ± SD)**	23.02 ± 3.37	20.16 ± 1.81	23.95 ± 1.82	27.75 ± 2.75	< 0.001
**TyG-BMI**_**2012 **_ **(M ± SD)**	195.83 ± 36.00	165.60 ± 15.77	202.49 ± 16.02	253.83 ± 28.83	< 0.001
**TyG-BMI**_**2015 **_ **(M ± SD)**	199.00 ± 36.22	167.01 ± 16.26	208.26 ± 16.35	254.69 ± 27.29	< 0.001
**Cumulative TyG‑BMI** **(M ± SD)**	592.24 ± 102.41	498.92 ± 40.85	616.12 ± 35.66	762.78 ± 63.12	< 0.001

### Clustering of changes in TyG-BMI from 2012 to 2015

**Fig. 2:**
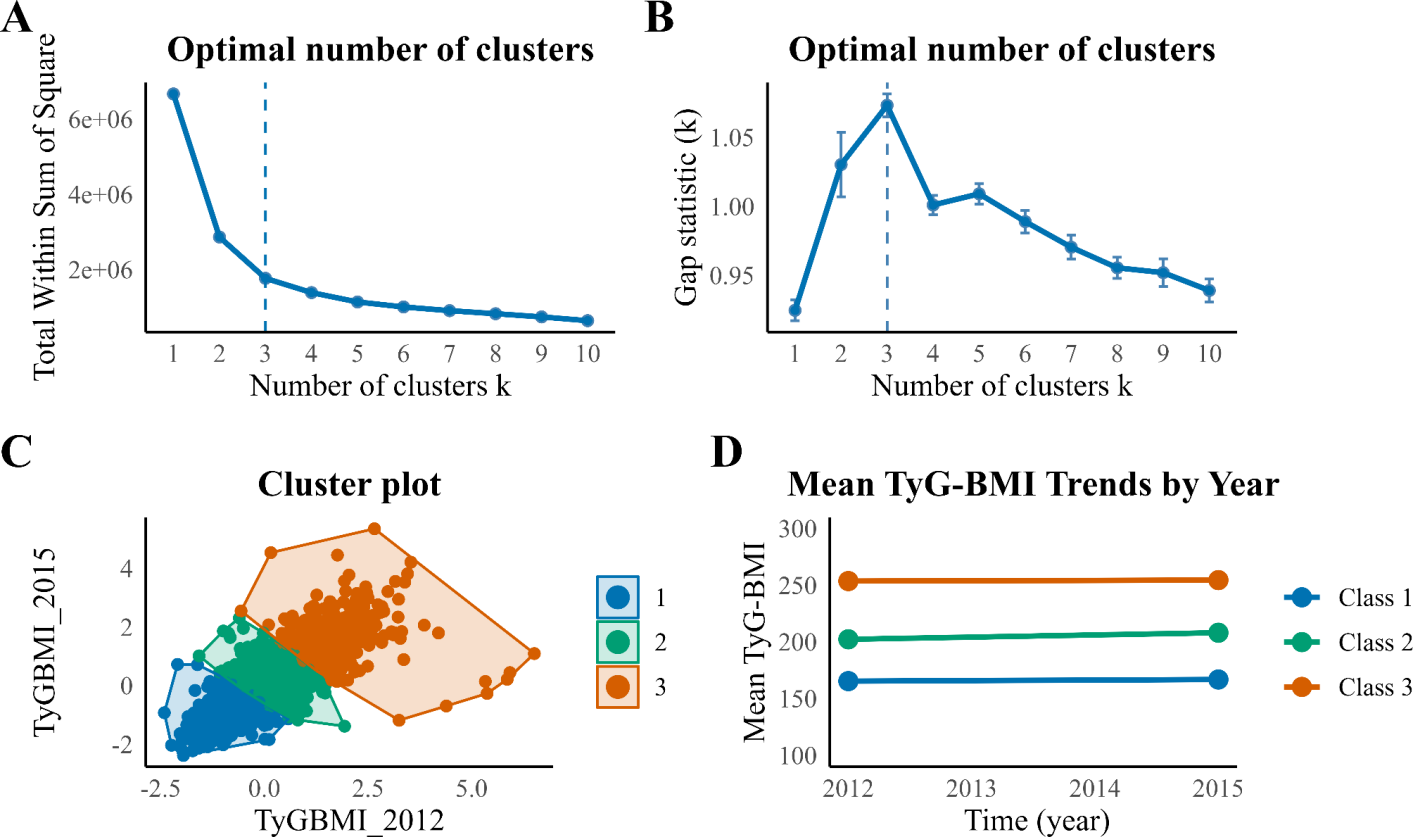
Clustering of the changes in the TyG‑BMI from 2012 to 2015. **(A-B)** Determine the optimal number of clusters for the changes in TyG-BMI using the Within-Cluster Sum of Squares (WSS) and Gap Statistics methods; **(C)** Clustering diagrams for TyG-BMI_2012_ and TyG-BMI_2015_; **(D)** Data visualization of mean TyG-BMI across different classes

The elbow method and the gap statistic were applied to determine the optimal number of clusters for changes in TyG-BMI (Fig. 2A and B). Based on the point where the rate of WSS reduction sharply leveled off and the maximum gap statistic was observed, the transition of TyG-BMI from 2012 to 2015 was categorized into three classes using K-means clustering (Fig. 2C). Specifically, the mean TyG-BMI value for class 1 (persistently low class) in 2012 and 2015 was 165.60 and 167.01, for class 2 (persistently medium class) was 202.49 and 208.26, and for class 3 (persistently high class) was 253.83 and 254.69 (Fig. 2D), respectively.

### Association of changes in TyG-BMI and cumulative TyG-BMI with incident hypertension

**Fig. 3:**
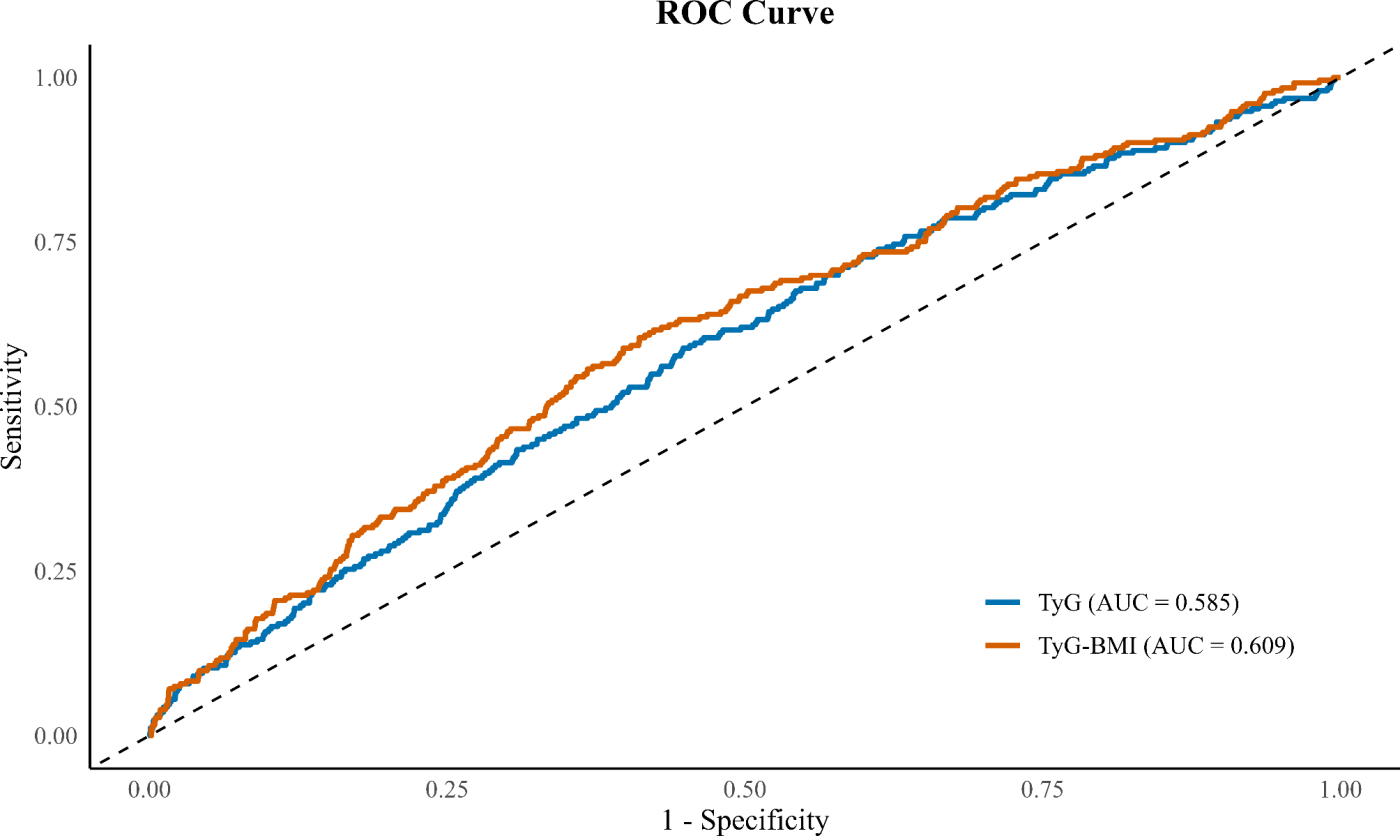
Receiver operating characteristic (ROC) curves for the prediction of hypertension based on baseline TyG-BMI and TyG

**Fig. 4:**
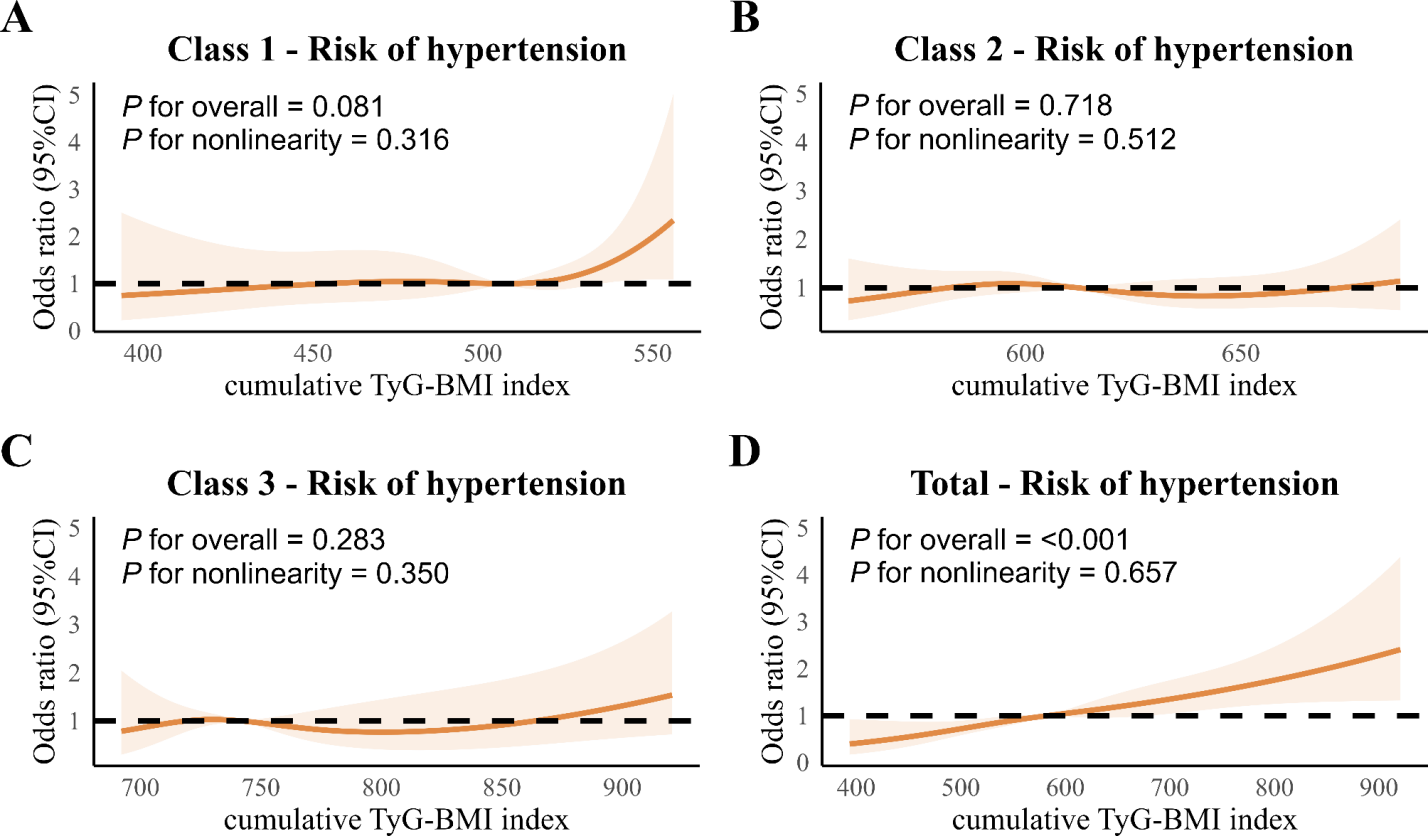
Cubic model of the association between different classes and cumulative TyG-BMI index after adjusting for age, sex, education level, marital status, residence, smoking, alcohol drinking, dyslipidemia, diabetes, heart disease, stroke, kidney disease, and CRP

During a 7-year follow-up period, 253 (9.9%) participants developed hypertension. As shown in Table 2, changes in TyG-BMI in class 2 and class 3 were associated with an increased risk of hypertension compared with class 1 across all four models. The HR (95% CI) was 1.50 (1.10–2.03) in class 2 and 2.35 (1.61–3.42) in class 3 in the fully adjusted model 3. Additionally, cumulative TyG-BMI in quartile 3 and 4 was linked to an elevated risk of hypertension across all four models compared with quartile 1. The HR (95% CI) was 1.75 (1.18–2.59) for quartile 3, and 2.15 (1.43–3.23) for quartile 4, respectively. Furthermore, ROC analyses indicated that TyG-BMI had significantly higher accuracy in predicting hypertension than TyG (AUC, 0.609 vs. 0.585; *P* = 0.023) **(**Fig. 3**)**. In the RCS model, there was a linear association between cumulative TyG-BMI and risk of hypertension in all subclasses of TyG-BMI and the total population (*P* for nonlinearity = 0.316, 0.512, 0.350, 0.657), while TyG-BMI was significantly associated with hypertension only in the total population (*P* for overall = < 0.001). The risk of hypertension was increased with each increase in the cumulative TyG-BMI above 581.6 (HR: 1.00, 95% CI: 0.99-1.00) (Fig. 4A and D).

**Table 2:** Different classes of TyG-BMI, cumulative TyG-BMI and risk of hypertension among participants

	Number of events/Total	Model 1		Model 2		Model 3	
		HR (95% CI)	*P* value	HR (95% CI)	*P* value	HR (95% CI)	*P* value
Changes in TyG-BMI							
Class 1	77/1050	1.0 (Ref)		1.0 (Ref)		1.0 (Ref)	
Class 2	112/1089	1.49 (1.11–2.01)	0.008	1.54 (1.14–2.07)	0.004	1.50 (1.10–2.03)	0.009
Class 3	64/358	2.33 (1.66–3.27)	< 0.001	2.48 (1.76–3.51)	< 0.001	2.35 (1.61–3.42)	< 0.001
Cumulative TyG‑BMI							
Quartile 1	43/641	1.0 (Ref)		1.0 (Ref)		1.0 (Ref)	
Quartile 2	58/640	1.42 (0.95–2.10)	0.085	1.48 (0.99–2.20)	0.055	1.46 (0.98–2.18)	0.062
Quartile 3	69/640	1.73 (1.18–2.55)	0.005	1.81 (1.23–2.67)	0.003	1.75 (1.18–2.59)	0.006
Quartile 4	83/640	2.16 (1.48–3.15)	< 0.001	2.32 (1.58–3.41)	< 0.001	2.15 (1.43–3.23)	< 0.001
*P* for trend			< 0.001		< 0.001		< 0.001

Model 1: Adjusted for age and sex

Model 2: Adjusted for age, sex, education level, marital status, residence, smoking, and alcohol drinking

Model 3: Adjusted for dyslipidemia, diabetes, heart disease, stroke, kidney disease, and CRP on the basis of model 2

### Association of changes in TyG-BMI and cumulative TyG-BMI with cumulative SBP and DBP

Table 3 presents the association between changes in TyG-BMI and cumulative TyG-BMI with cumulative SBP and DBP, adjusted for various potential confounders (Model 3). For changes in TyG-BMI, compared to class 1 (reference), class 2 showed a significant increase in cumulative SBP (7.70 mmHg, 95% CI: 5.18–10.21) and cumulative DBP (6.53 mmHg, 95% CI: 4.68–8.38), while class 3 exhibited larger increases in cumulative SBP (14.10 mmHg, 95% CI: 10.56–17.64) and cumulative DBP (12.64 mmHg, 95% CI: 10.03–15.25). For cumulative TyG-BMI, quartile 1 is the reference group. Compared to quartile 1, quartile 2 was associated with a significant increase in cumulative SBP (3.99 mmHg, 95% CI: 0.88–7.11) and cumulative DBP (2.74 mmHg, 95% CI: 0.45–5.02). Quartile 3 showed significant increases in cumulative SBP (8.32 mmHg, 95% CI: 5.09–11.54) and cumulative DBP (7.13 mmHg, 95% CI: 4.76–9.49). Quartile 4 had the highest increases in cumulative SBP (13.15 mmHg, 95% CI: 9.70–16.60) and cumulative DBP (12.20 mmHg, 95% CI: 9.67–14.74).

**Table 3:** Association of changes in TyG-BMI, cumulative TyG-BMI and cumulative SBP and cumulative DBP

	Cumulative SBP (mmHg)		Cumulative DBP (mmHg)	
	β* (95% CI)	*P* value	β* (95% CI)	*P* value
Changes in TyG-BMI				
Class 1	1.0 (Ref)		1.0 (Ref)	
Class 2	7.70 (5.18–10.21)	< 0.001	6.53 (4.68–8.38)	< 0.001
Class 3	14.10 (10.56–17.64)	< 0.001	12.64 (10.03–15.25)	< 0.001
Cumulative TyG-BMI				
Quartile 1	1.0 (Ref)		1.0 (Ref)	
Quartile 2	3.99 (0.88–7.11)	0.012	2.74 (0.45–5.02)	0.019
Quartile 3	8.32 (5.09–11.54)	< 0.001	7.13 (4.76–9.49)	< 0.001
Quartile 4	13.15 (9.70–16.60)	< 0.001	12.20 (9.67–14.74)	< 0.001

* Adjusted for age, sex, education level, marital status, residence, smoking, alcohol drinking, dyslipidemia, diabetes, heart disease, stroke, kidney disease, and CRP

### Subgroup analysis

The subgroup analysis (Tables 4 and 5**)** indicated no significant interactions between changes in TyG-BMI, cumulative TyG-BMI and the subgroup variables after adjusting for potential confounders. Results consistently showed an association between changes in TyG-BMI, cumulative TyG-BMI, and hypertension.

**Table 4:** Subgroup analysis for the association (HR, 95% CI) between changes in TyG-BMI and risk for hypertension

Subgroup	Number of events/Total	Class 1	Class 2*	Class 3*	*P* for interaction
**Age (years)**					0.385
< 60	151/1658	1.0 (Ref)	1.61 (1.11–2.55)	2.19 (1.34–3.59)	
≥ 60	102/903	1.0 (Ref)	1.29 (0.81–2.05)	2.89 (1.59–5.28)	
**Sex**					0.259
Male	109/1142	1.0 (Ref)	1.49 (0.96–2.30)	1.55 (0.82–2.95)	
Female	144/1419	1.0 (Ref)	1.52 (0.98–2.33)	3.01 (1.85–4.90)	
**Education level**					0.848
Illiterate	83/660	1.0 (Ref)	1.22 (0.73–2.04)	2.14 (1.09–4.18)	
Primary school	92/1058	1.0 (Ref)	1.79 (1.07–3.01)	3.20 (1.71-6.00)	
Middle school or above	78/843	1.0 (Ref)	1.64 (0.91–2.93)	2.04 (1.02–4.07)	
**Marital status**					0.943
Married	230/2371	1.0 (Ref)	1.48 (1.07–2.04)	2.35 (1.59–3.49)	
Single or other	23/190	1.0 (Ref)	1.48 (0.54–4.09)	2.27 (0.53–9.82)	
**Residence**					0.207
Rural	177/1775	1.0 (Ref)	1.45 (1.01–2.07)	2.90 (1.86–4.52)	
Urban	76/786	1.0 (Ref)	1.61 (0.88–2.95)	1.58 (0.77–3.24)	
**Smoking**					0.413
Smoking	98/959	1.0 (Ref)	1.44 (0.91–2.29)	1.53 (0.80–2.92)	
Non-smoking	155/1602	1.0 (Ref)	1.56 (1.03–2.36)	2.91 (1.80–4.71)	
**Alcohol drinking**					0.262
> 1 times per month	62/631	1.0 (Ref)	1.83 (1.03–3.26)	1.26 (0.53–2.98)	
< 1 times per month	21/228	1.0 (Ref)	0.81 (0.27–2.40)	1.25 (0.37–4.21)	
Non-consumer	170/1702	1.0 (Ref)	1.64 (1.12–2.42)	3.23 (2.04–5.13)	
**Dyslipidemia**					0.727
Yes	124/1064	1.0 (Ref)	1.69 (0.99–2.87)	2.34 (1.34–4.08)	
No	129/1497	1.0 (Ref)	1.46 (0.99–2.14)	2.64 (1.50–4.68)	
**Diabetes**					0.231
Yes	34/307	1.0 (Ref)	0.62 (0.23–1.67)	1.73 (0.66–4.52)	
No	219/2254	1.0 (Ref)	1.61 (1.16–2.22)	2.36 (1.56–3.56)	
**Heart disease**					0.197
Yes	26/194	1.0 (Ref)	2.06 (0.60–7.10)	5.86 (1.63–21.09)	
No	227/2367	1.0 (Ref)	1.45 (1.06–1.99)	2.12 (1.42–3.17)	
**Stroke**					0.238
Yes	4/29	1.0 (Ref)	NA	NA	
No	249/2532	1.0 (Ref)	1.48 (1.09–2.01)	2.32 (1.58–3.39)	
**Kidney disease**					0.063
Yes	23/184	1.0 (Ref)	0.65 (0.21–2.01)	2.97 (0.91–9.68)	
No	230/2377	1.0 (Ref)	1.63 (1.18–2.25)	2.32 (1.55–3.46)	

*: Adjusted for age, sex, education level, marital status, residence, smoking, and alcohol drinking, dyslipidemia, diabetes, heart disease, stroke, kidney disease, and CRP, except in the corresponding group

**Table 5:** Subgroup analysis for the association (HR, 95% CI) between cumulative TyG-BMI and risk for hypertension

Subgroup	Quartile 1	Quartile 2*	Quartile 3*	Quartile 4*	*P* for trend	*P* for interaction
**Age (years)**						0.395
< 60	1.0 (Ref)	1.25 (0.69–2.24)	1.99 (1.15–3.43)	2.13 (1.22–3.71)	< 0.001	
≥ 60	1.0 (Ref)	1.73 (1.00-2.98)	1.39 (0.76–2.54)	2.25 (1.20–4.23)	0.003	
**Sex**						0.351
Male	1.0 (Ref)	1.88 (1.10–3.22)	1.62 (0.88–2.96)	2.14 (1.13–4.04)	0.012	
Female	1.0 (Ref)	1.07 (0.58–1.96)	1.77 (1.04–3.01)	2.13 (1.24–3.66)	< 0.001	
**Education level**						0.679
Illiterate	1.0 (Ref)	1.52 (0.79–2.95)	1.71 (0.90–3.26)	1.76 (0.86–3.59)	0.010	
Primary school	1.0 (Ref)	1.19 (0.64–2.22)	1.32 (0.68–2.56)	2.37 (1.23–4.56)	0.002	
Middle school or above	1.0 (Ref)	2.14 (0.87–5.24)	2.91 (1.23–6.88)	2.99 (1.25–7.15)	0.007	
**Marital status**						0.466
Married	1.0 (Ref)	1.34 (0.88–2.04)	1.66 (1.09–2.51)	2.12 (1.39–3.24)	< 0.001	
Single or other	1.0 (Ref)	3.38 (0.94–12.13)	2.39 (0.66–8.72)	1.93 (0.40–9.40)	0.593	
**Residence**						0.659
Rural	1.0 (Ref)	1.56 (0.99–2.45)	1.73 (1.09–2.74)	2.46 (1.53–3.95)	< 0.001	
Urban	1.0 (Ref)	1.12 (0.48–2.61)	1.67 (0.76–3.70)	1.50 (0.67–3.36)	0.156	
**Smoking**						0.116
Smoking	1.0 (Ref)	2.26 (1.27–4.01)	1.73 (0.90–3.34)	2.24 (1.14–4.39)	0.005	
Non-smoking	1.0 (Ref)	0.96 (0.54–1.69)	1.64 (0.99–2.70)	1.97 (1.18–3.30)	< 0.001	
**Alcohol drinking**						0.994
> 1 times per month	1.0 (Ref)	1.78 (0.84–3.76)	1.96 (0.89–4.35)	2.20 (0.97–5.02)	0.069	
< 1 times per month	1.0 (Ref)	1.21 (0.25–5.74)	1.54 (0.41–5.85)	1.14 (0.26–5.01)	0.943	
Non-consumer	1.0 (Ref)	1.36 (0.82–2.26)	1.78 (1.09–2.91)	2.47 (1.49–4.09)	< 0.001	
**Dyslipidemia**						0.638
Yes	1.0 (Ref)	2.32 (1.02–5.29)	2.32 (1.06–5.09)	3.13 (1.47–6.67)	< 0.001	
No	1.0 (Ref)	1.27 (0.80–2.03)	1.75 (1.08–2.83)	1.85 (1.04–3.28)	0.005	
**Diabetes**						0.179
Yes	1.0 (Ref)	1.29 (0.38–4.39)	0.42 (0.09–1.87)	1.62 (0.48–5.50)	0.061	
No	1.0 (Ref)	1.44 (0.94–2.20)	1.91 (1.26–2.89)	2.13 (1.38–3.29)	< 0.001	
**Heart disease**						0.409
Yes	1.0 (Ref)	1.19 (0.23–6.24)	2.95 (0.68–12.72)	2.99 (0.73–12.18)	0.011	
No	1.0 (Ref)	1.51 (1.00-2.28)	1.69 (1.11–2.55)	2.13 (1.38–3.28)	< 0.001	
**Stroke**						0.334
Yes	1.0 (Ref)	NA	NA	NA	NA	
No	1.0 (Ref)	1.44 (0.97–2.15)	1.70 (1.14–2.54)	2.11 (1.40–3.19)	< 0.001	
**Kidney disease**						0.509
Yes	1.0 (Ref)	2.65 (0.72–9.69)	1.00 (0.22–4.52)	3.57 (0.99–12.83)	0.040	
No	1.0 (Ref)	1.44 (0.95–2.20)	1.83 (1.21–2.78)	2.10 (1.36–3.24)	< 0.001	

*: Adjusted for age, sex, education level, marital status, residence, smoking, alcohol drinking, dyslipidemia, diabetes, heart disease, stroke, kidney disease, and CRP, except in the corresponding group

## Discussion

This study investigated the relationship of changes in TyG-BMI and cumulative TyG-BMI with the risk of hypertension and cumulative SBP and DBP among middle-aged and elderly Chinese adults. Our findings demonstrate that both changes in TyG-BMI and cumulative TyG-BMI were significantly associated with an increased risk of developing hypertension, along with higher cumulative SBP and DBP, and the relationship between cumulative TyG-BMI and hypertension was observed to be linear. Additionally, baseline TyG-BMI had greater accuracy in predicting hypertension compared with TyG. The findings of this study suggest that monitoring changes in TyG-BMI and cumulative TyG-BMI could be crucial in identifying individuals at higher risk of developing hypertension.

Our results are consistent with other studies that have shown a relationship between the TyG-BMI index and hypertension. In a cross-sectional study of 92,545 participants, Deng et al. found a linear association between TyG-BMI and hypertension (*P* for nonlinearity = 0.062) [11]. Another cross-sectional study with 16,834 participants in Beijing, China showed that the TyG-BMI index had a stronger association with hypertension compared to the TyG index. In addition, the TyG-BMI index (AUC: 0.658, 95% CI: 0.650–0.666) showed a higher predictive value for hypertension than the TyG index (AUC: 0.614, 95% CI: 0.605–0.622) [12]. A population-based retrospective study of 214,493 participants in East Asian populations showed that TyG-BMI was linearly correlated with both hypertension and pre-hypertension, and TyG-BMI was independently correlated with hypertension [25].

Limited studies have shown that changes in the TyG-BMI index or cumulative TyG-BMI index were associated with cardiovascular events. A cohort study of 4,583 participants in CHARLS showed that changes in TyG‑BMI and cumulative TyG-BMI were independently associated with stroke risk. The association between cumulative TyG‑BMI and stroke risk was nonlinear (*P* for association = 0.017, *P* for nonlinearity = 0.012) [20]. Li et al. found a positive and linear association of cumulative TyG-BMI with cardiovascular disease (CVD) incidence (*P* for overall = 0.038, *P* for nonlinearity = 0.436) [26]. Another study of CHARLS examined changes in TyG-BMI from 2011 to 2015 and reported that participants in the highest tertile of changes in TyG-BMI had a higher risk of hypertension (OR: 1.26, 95% CI: 1.07–1.48) compared to those in the lowest tertile [15]. However, the outcome was only measured in wave 3, and this may compromise the causal relationship assessment. Additionally, it only measured changes in TyG-BMI over a 4-year period and did not investigate the effects on blood pressure. In contrast, our study not only considered newly diagnosed hypertension cases during a 7-year period, but also examined the cumulative blood pressure, enhancing the clarity of the causal link. Furthermore, we employed a machine learning approach (k-means clustering) for a more objective and systematic classification of changes in TyG-BMI.

Our RCS model showed a linear association between the cumulative TyG-BMI and hypertension, although the association was not significant within three TyG-BMI classes. The small number of hypertension cases in each class may have limited our ability to detect such an association. In addition, subgroup analysis revealed that none of the subgroups (such as age, sex, education level, residence, and history of dyslipidemia, or diabetes) showed significant modification on the association between changes in TyG-BMI and cumulative TyG-BMI and the incidence of hypertension, emphasizing the relevance of our findings to a wide range of individuals.

TyG-BMI could act as an agent of insulin resistance, with elevated levels correlating with increased hypertension risk, potentially driven by various mechanisms. Firstly, IR disrupts renal sodium handling, leading to sodium retention and expansion of extracellular fluid volume, consequently elevating blood pressure [27, 28]. Furthermore, IR undermines the kidney’s capacity to regulate blood pressure via the pressure-natriuresis relationship [29]. Secondly, IR fosters endothelial dysfunction by diminishing nitric oxide availability, escalating oxidative stress, and promoting inflammation. This dysfunction impairs vasodilation, thereby heightening vascular resistance and hypertension [30, 31]. Lastly, higher TyG-BMI often accompanies dyslipidemia, characterized by elevated triglycerides, exacerbating vascular injury and atherosclerosis. These lipid abnormalities compromise vascular compliance, exacerbating arterial stiffness and elevating systolic blood pressure [32–34].

The strengths of this study include the prospective cohort design which allows for the assessment of temporal relationships between changes in TyG-BMI and the development of hypertension. Furthermore, we took into account a variety of potential confounders, including demographic factors, lifestyle habits and pre-existing health conditions. The use of k-means clustering to categorize changes in TyG-BMI, as well as multivariate logistic regression and restricted cubic spline regression provide robust analytic frameworks for evaluating the data. These methods increase the reliability of the results. However, our study has some limitations. First, the follow-up period of seven years, while substantial, may not fully capture the long-term effects of changes in TyG-BMI on hypertension risk. Thus, future research with longer follow-up periods may provide more comprehensive insights into the temporal dynamics of this relationship. Second, despite extensive adjustments, the possibility of residual confounding may exist. Unmeasured factors, such as genetic predispositions or other lifestyle variables that were not taken into account, could influence the observed associations. Third, TyG-BMI in CHARLS was measured only at two time points (2012 and 2015), this might not capture short-term fluctuation or the full trajectory of metabolic changes over time, thus affecting the relationship between TyG-BMI and hypertension risk. Fourth, hypertension was defined based on self-reported data, medical diagnoses, or measurements of SBP and DBP. This may result in some misclassification, particularly for participants who were not diagnosed or who did not accurately report their condition. Fifth, although the results of the study are applicable to the middle-aged and elderly Chinese population, they may not be directly generalizable to other ethnic or age groups.

## Conclusions

The study demonstrates a significant association between changes in TyG-BMI and cumulative TyG-BMI with the risk of hypertension in middle-aged and older Chinese adults. Future research among diverse populations and with longer follow-up periods are warranted to confirm the long-term effects of changes in TyG-BMI and cumulative TyG-BMI on hypertension risk.

## Data Availability

Researchers may obtain the datasets after sending a data user agreement to the CHARLS team. https://charls.pku.edu.cn/.
